# Enhanced pathogenicity by up-regulation of A20 after avian leukemia subgroup a virus infection

**DOI:** 10.3389/fvets.2022.1031480

**Published:** 2022-11-14

**Authors:** Xueyang Chen, Xingming Wang, Yuxin Yang, Chun Fang, Jing Liu, Xiongyan Liang, Yuying Yang

**Affiliations:** ^1^College of Animal Science, Yangtze University, Jingzhou, China; ^2^College of Agriculture, Yangtze University, Jingzhou, China

**Keywords:** ALV-A, chicken, A20, recombinant adenovirus, pathogenic

## Abstract

Avian leukemia virus subgroup A (ALV-A) infection slows chicken growth, immunosuppression, and tumor occurrence, causing economic loss to the poultry industry. According to previous findings, A20 has a dual role in promoting and inhibiting tumor formation but has rarely been studied in avians. In this study, A20 overexpression and shRNA interference recombinant adenoviruses were constructed and inoculated into chicken embryos, and ALV-A (rHB2015012) was inoculated into 1-day-old chicks. Analysis of body weight, organ index, detoxification, antibody production, organ toxin load, and Pathological observation revealed that A20 overexpression could enhance ALV-A pathogenicity. This study lays the foundation for subsequent exploration of the A20-mediated tumorigenic mechanism of ALV-A.

## Introduction

Avian leukemia (AL) is a neoplastic disease caused by avian leukemia virus (ALVs), which is classified into the A-K 11 subgroup of the retrovirus family (1–3). Avian leukemia virus subgroup A (ALV-A) is an exogenous virus that causes immunosuppression, slow growth, severe pathology, tumorigenesis, and death in infected chickens (1, 4). In some European and American countries, it took several years to eradicate ALV-A. However, there are still reports on ALV-A strain prevalence (5–9), which causes economic losses to the poultry industry (2, 10). Currently, no vaccines or drugs are available to prevent and control ALV-A infections.

A20 was discovered in 1990 by stimulating human umbilical epithelial cells with tumor necrosis factor-alpha (TNF-α). Hence, A20 is known as tumor necrosis factor α-induced protein 3 (TNFAIP3) (11), which negatively regulates the NF-κB signaling pathway (12). A20 is a ubiquitin-editing enzyme that plays diverse roles in various diseases, such as periodontitis, viral myocarditis, and autoimmune disease (13–17). Many reports on A20 in cancer mention that it can be used as a suppressor or promoter in this disease, depending on its expression (18–23). A20 upregulation alleviates LPS-induced activation of the NF-κB signaling pathway and generation of inflammatory responses in avian chicken intestinal epithelial cells (24). Moreover, there have been no reports on A20 role in avian tumorigenic diseases.

Previous research has found that A20 transcription was upregulated after ALV-A infection (HB2015012) in laying hens (Supplementary Figure 1), implying that A20 may be involved in ALV-A infection or tumorigenesis. Therefore, in this study, we constructed A20-overexpressing and shRNA recombinant adenoviruses and inoculated chicken embryos to evaluate the role of A20 in ALV-A infection. These findings may help establish the tumorigenic mechanism of ALV-A mediated by A20.

## Materials and methods

### Cells, viruses, plasmids, and animal

HEK-293T cells (SC21091507, Hunan Fenghui Biotechnology Co., Ltd, China) were cultured in Dulbecco′s modified Eagle's medium (C11995500, Gibco, USA) supplemented with 10% fetal bovine serum (FBS, Gemini, 900–108, USA), 100 μg/mL streptomycin, and 100 U/mL penicillin at 37 °C in an atmosphere of 5% CO_2_. The virus strain rHB2015012 (GeneBank ID: KY612442) was rescued and stored in our laboratory (25). The plasmids pShuttle-IRES-hrGFP-2 (240082, Agilent, USA), pshuttle-H1 (BioVector-922362, NTCC, China), pAd-Easy-1 (240005, Agilent, USA), E. coli BJ5183 (200154, Agilent, USA), and E. coli DH5α were maintained in our laboratory. Jingfen No. 1, 4-day-old chicken embryos were purchased from a hatchery in Hubei (China).

### A20 recombinant adenovirus construction

The overexpressed adenovirus primers were designed according to NCBI sequences for chicken A20 (XR_005848478). A20 was amplified using PCR and cloned into the vector pShuttle-IRES-hrGFP-2. Two shRNAs were designed using an online website (http://rnaidesigner.thermofisher.com/rnaiexpress/) and cloned into the vector pShuttle-H1 after annealing. The primer sequences are listed in Table 1. The recombinant adenoviruses were constructed according to manufacturer's instructions (26). The constructed A20 adenovirus was inoculated into 10-day-old chicken embryos (10^8^ TCID_50_/egg). The liver and kidneys were harvested 20 days after infection and treated with NP-40 cell lysate (P0013F, Beyotime, China). It was analyzed using Western blotting as previously described (27). The antibodies and dilutions used for Western blotting were as follows: anti-ALV-A envelope glycoprotein-specific antibody (prepared in our laboratory, 1:1,000), rabbit Anti-A20 antibody (GB112182, Servicebio, China, 1:1,000), mouse anti-GAPDH monoclonal antibody (60004, Proteintech, USA, 1:5,000), HRP-conjugated goat anti-rabbit IgG (D110058, Sangon Biotech, China, 1:5,000), and HRP-conjugated goat anti-mouse IgG (D110087, Sangon Biotech, China, 1:5,000).

**Table 1:** A20 recombinant adenovirus construction primers.

**Plasmids**	**Primers**	**Sequences (5'-3')**
pShuttle-IRES-hrGFP-2-A20	F	CACTAGTGATATCCGATCGGTCGACATGGCTGGCCAACACATC
	R	CTGGAACGTCATATGGGTACTCGAGGCCGTAGATCTGTTTGAACTGG
pShuttle-H1-A20-shRNA	F	TCGACGCTTTGTATCAGAGCAATATGTTCAAGAGACATATTGCTCTGATACAAAGCTTTTTTC
	R	TCGAGAAAAAAGCTTTGTATCAGAGCAATATGTCTCTTGAACATATTGCTCTGATACAAAGCG

### The effect of A20 on the replication of ALV-A *in vitro*

The constructed A20 adenovirus was used to infect DF-1 cells at a density of 80% in a 6-well cell culture plate (MOI = 100). After 24 h, 10^3^ TCID_50_ of ALV-A rHB2015012 virus solution was added to each well of the cells, and only ALV-A rHB2015012 was used as a control. Cells were harvested on days 1, 2, 3, and 4 of infection. Total mRNA was extracted using an RNA extraction kit (TSP413, Tsingke, China), and reverse transcription was performed using a HiScripIII 1st Strand cDNA Synthesis Kit (R312, Vazyme, China). Reverse transfection was carried out according to the manufacturer's instructions, and qPCR was performed using ChamQ Universal SYBR qPCR Master Mix (Q711, Vazyme, China) according to the manufacturer's instructions. Chicken β-actin was used as the endogenous control for A20 and ALV-A p27. qPCR was performed on an ABI 7,500 qPCR system (USA). The relative expression levels of each gene were calculated and normalized using the 2^−ΔΔCt^ method. The qPCR primer sequences used are listed in Table 2.

**Table 2:** Primers for q-PCR.

**Gene**	**Primers**	**Sequences (5'-3')**	**Product size (bp)**
β-actin	F	CAACACACTGCTGTCTGGTGGTA	204
	R	ATCGTACTCCTGCTTGCTGATCC	
A20	F	TGGGGCTCGAAACAGACTTC	222
	R	TTGTCGTAGCCGAGCACAAT	
p27	F	CCGGGGGAATTGGTTGCTAT	225
	R	ATCTGGCTGTGACTTCTGCC	

### Animal grouping and infectivity testing

**Figure 1:**
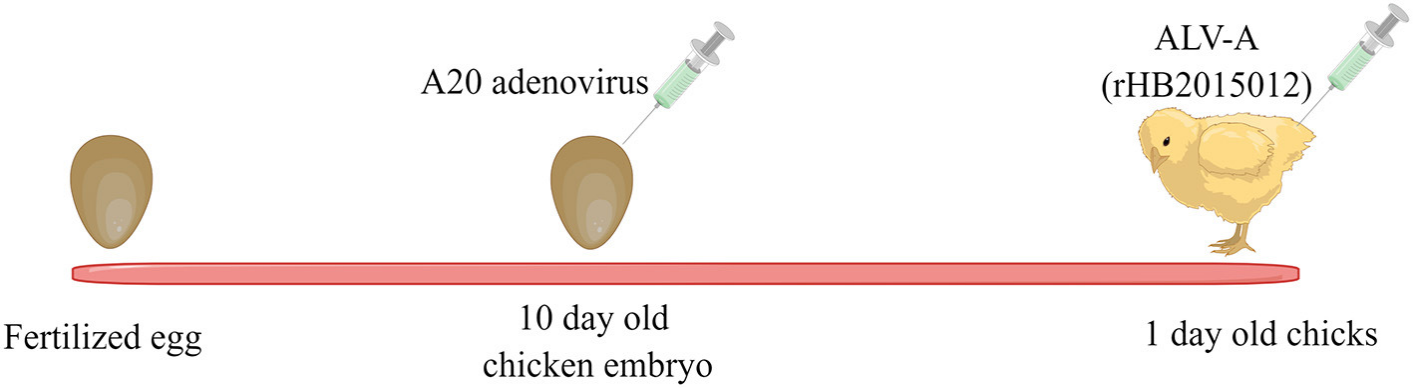
A20 recombinant adenovirus and ALV-A vaccination schedule. Ten-day-old chicken embryos were inoculated with A20 recombinant adenovirus, and one-day-old chickens were inoculated with ALV-A.

Eighty chick embryos (not SPF) were divided into four groups, with 20 in each group (at least 18 eggs were successfully hatched in each group). The PBS control group was negative. The positive group was inoculated only with ALV-A (rHB2015012) at 1-day-old chicks, and each chick was injected with 10^4^ TCID_50_ ALV-A virus (diluted to 0.2 mL with PBS) through the legs. The overexpression and shRNA groups were inoculated with A20 recombinant adenovirus in the allantoic cavity of 10-day-old chicken embryos, with 2 × 10^8^ TCID_50_ (diluted to 0.2 mL with PBS) of virus per chicken embryo; meanwhile, negative and positive groups were injected with 0.2 mL PBS in the allantoic cavity of each chick embryo. In the positive, overexpression, and shRNA groups, each 1-day-old chick was inoculated with 10^4^ TCID_50_ (diluted to 0.2 mL with PBS) ALV-A (rHB2015012) through the leg muscle, while the negative group was inoculated with 0.2 mL PBS through the leg. A schematic diagram for the virus inoculation procedure is displayed in Figure 1.

Body weight was measured 1, 3, 5, 7, 11, 14, 21, 28, and 35 days after infection. Blood and anal swabs were collected for virus and antibody testing on days 7, 14, 21, 28, and 35 post-infection. Three chickens were randomly selected for euthanasia and autopsy at 7, 14, 21, 28, and 35 days after infection. The heart, liver, spleen, lungs, kidneys, and bursa were collected to calculate the organ index (organ weight/body weight). The liver, spleen, and kidney were collected for viral load determination and preparation of pathological sections.

### Viremia and shed virus detection

Genomic DNA was extracted from the collected blood using a blood DNA extraction kit (D1800, Solarbio, China). ALV-A infection was detected using ALV-A/J/K multiplex PCR with the primers listed in Table 3.

**Table 3:** ALV-A/J/K multiplex PCR primers.

**Primers**	**Sequences (5'-3')**	**Product size (bp)**
ALV-A/J/K-F	CGGAGAAGACACCCTTGCT	
ALV-AR	GCATTGCCACAGCGGTACTG	715
ALV-JR	CGAACCAAAGGTAACACACG	422
ALV-KR	TTGCGGCCTGGACCAATC	535

ALV-A detection in the collected chicken serum (10 times dilution with PBS) and anal swabs was performed using a p27 group-specific antigen ELISA detection kit (99-09254, IDEXX, USA). According to the manufacturer's instructions, if S/P is > 0.2, the sample is positive; otherwise, it is negative. S/P = (sample absorbance value-negative control absorbance value)/(positive control absorbance value-negative control absorbance value).

### Anti-ALV-A and anti-p27 antibody detection

Purified ALV-A Gp85 protein (2 μg/well) and P27 protein (2 μg/well) were coated in a 96-well microtiter plate and incubated at 4 °C for 16–18 h. The ELISA plate was washed thrice with PBS containing 0.05% Tween-20 (PBST). Skim milk diluted in 5% PBST was blocked at 37 °C for 2 h and washed three times with PBST. A total of 100 μL test serum was added, and the negative serum was diluted in equal proportions in different wells. The samples were then incubated for 2 h and washed thrice with PBST. A 100 μL of HRP-conjugated goat anti-chicken IgG (D110205, Sangon Biotech, China) was added and diluted 5,000 times with 5% skim milk to each well, followed by incubation for 1 h and washing (in triplicate) with PBST. Chromogenic analysis was performed using a TMB Kit (PA107, Tiangen, China) following the manufacturer's instructions. If P/N > 2, the sample is positive; otherwise, the sample is negative. P/N = sample absorbance value-negative control absorbance value.

### Viral load determination in infected chicken organs

Total mRNA was extracted from the liver, spleen, and kidney using an RNA extraction kit, and reverse transcription was performed using the HiScript III 1st Strand cDNA Synthesis Kit. β-actin, A20, and p27 transcript levels were detected using qPCR, and each gene was analyzed using the 2^−ΔΔCt^ method.

### Relative expression of ALV-A Gp85 in infected chickens

The harvested liver, spleen, and kidney tissues were treated with NP-40 cell lysates. The relative expression of ALV-A Gp85 was determined using Western blotting, as previously described. Anti-ALV-A envelope glycoprotein Gp85 specific antibody was prepared in our laboratory (1:1,000 dilution).

### Statistical analysis

GraphPad Prism 6 (https://www.graphpad.com/) was used for statistical data analysis. The data are presented as means ± standard error of the mean (SEM). The data differences were evaluated using the Student′s *t*-test or one-way ANOVA test. *P* < 0.05 was considered statistically significant (^*^ *P* ≤ 0.05, ^**^P ≤ 0.01, and ^***^P ≤ 0.001).

## Results

### A20 recombinant adenovirus construction

**Figure 2:**
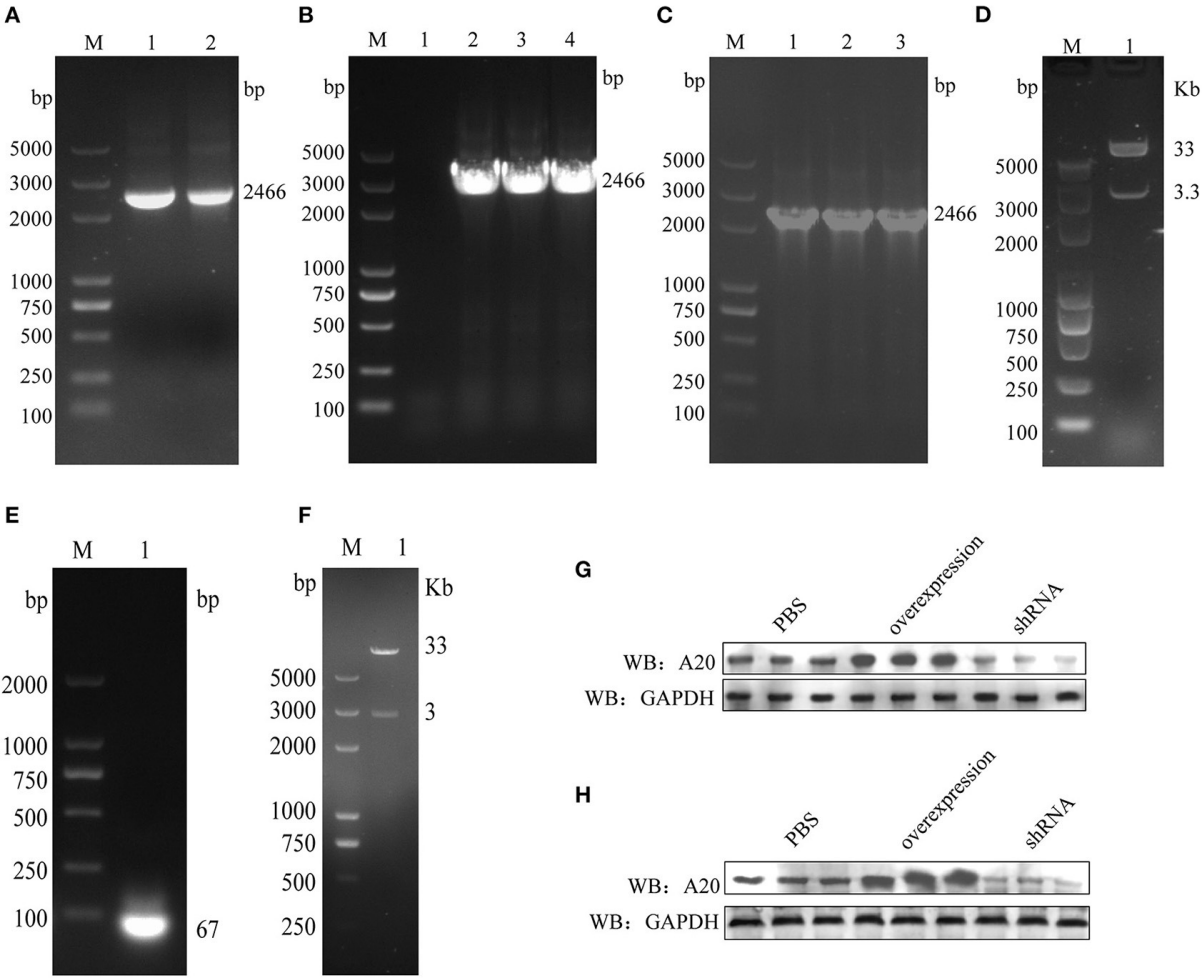
Constructing A20 recombinant adenovirus. **(A)** A20 gene amplification. **(B)** Identifying pshuttle-IRES-hrGFP2-A20 positive colonies. **(C)** Identifying Ad-pshuttle-IRES-hrGFP2-A20 positive colonies. **(D)** The plasmid Ad-pshuttle-IRES-hrGFP2-A20 has been digested with restriction endonuclease PacI. **(E)** A20 single-stranded shRNA anneals to double-stranded. **(F)** The plasmid Ad-pshuttle-H1-A20 shRNA is digested with restriction endonuclease PacI. **(G)** The effect of A20 adenovirus in the chicken embryo kidney. **(H)** The effect of A20 adenovirus in the chicken embryo liver.

The A20 gene was amplified by PCR to obtain the expected fragment size (2,466 bp, Figure 2A) and cloned into the vector pshuttle-IRES-hrGFP2 (Figure 2B). pshuttle-IRES-hrGFP2-A20 was linearized with restriction endonuclease *Pme*I and transformed into BJ5183 containing the backbone plasmid pAdEasy-1 for natural recombination (Figure 2C). The plasmid Ad-pshuttle-IRES-hrGFP2-A20 was digested with the restriction enzyme *Pac*I (Figure 2D), and the largest fragment was recovered and transfected into HEK293 cells. After most of the HEK293 cells fell off, the culture supernatant and cells were collected, frozen and thawed three times, and stored at −80 °C. Synthetic single-stranded shRNA was annealed with annealing buffer for DNA Oligos (D0251, Beyotime, China) and cloned into pshuttle-H1 (Figure 2E); the construction process was the same as A20 overexpressing adenovirus (Figure 2F). The constructed A20 adenovirus can overexpress and interfere with A20 expression (Figures 2G,H).

### A20 promotes viral replication *in vitro*

**Figure 3:**
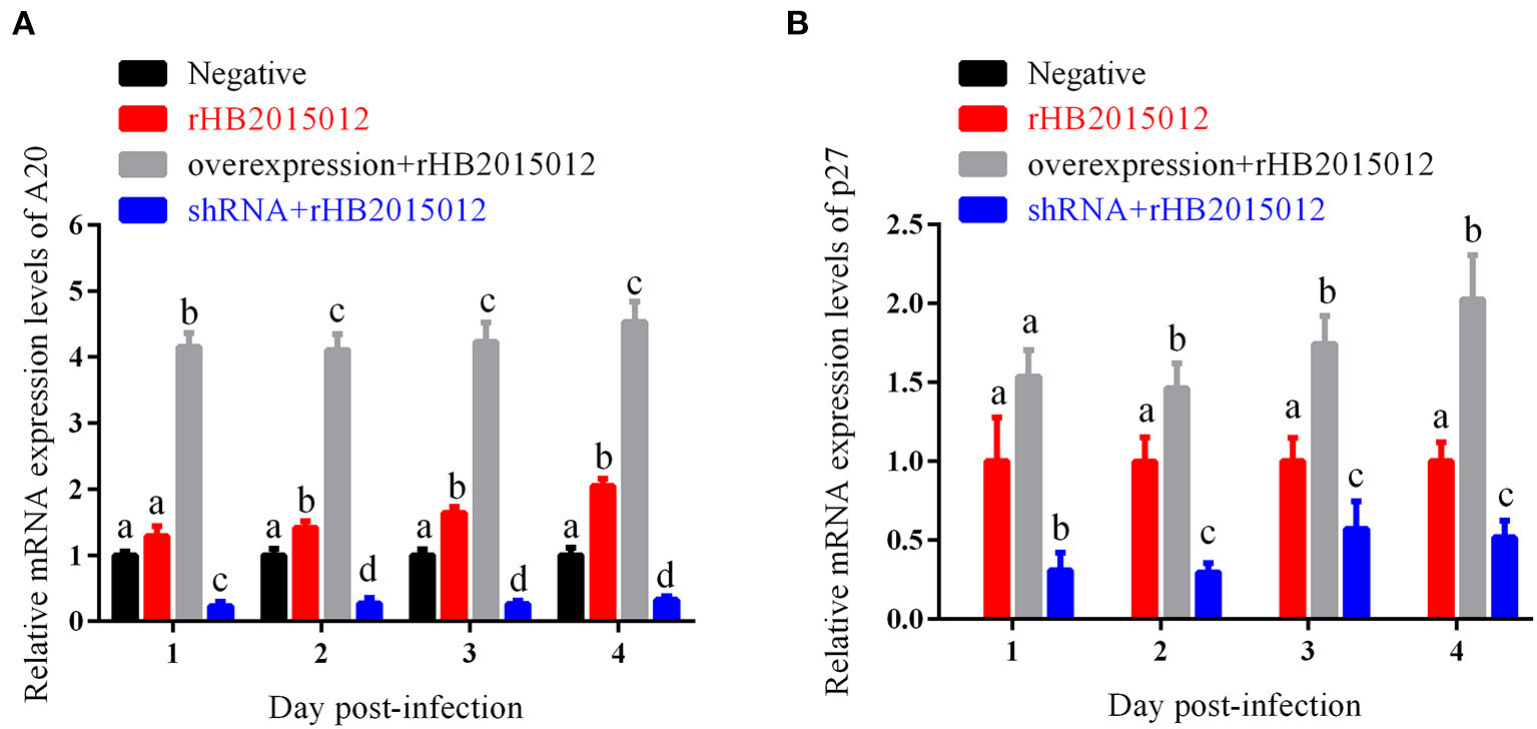
Effects of A20 on ALV-A rHB2015012 *in vitro* replication. **(A)** A20 recombinant adenoviruses capable of overexpressing and interfering with A20 production in DF-1 cells. **(B)** A20 promotes the replication of ALV-A rHB2015012 in DF-1 cells. a, b, c, and d indicate differences, with the same letter indicating no difference and different letters indicating a difference.

Overexpression and perturbation of A20 protein production in DF-1 cells by A20 recombinant adenovirus infection. It was revealed that the constructed adenovirus could overexpress and interfere with the expression of A20 protein (Figure 3A). It has also been shown that A20 promotes the replication of ALV-A rHB2015012 and that A20 protein expression was disturbed and inhibited the replication of ALV-A rHB2015012 (Figure 3B).

### A20 overexpression caused slow growth

**Figure 4:**
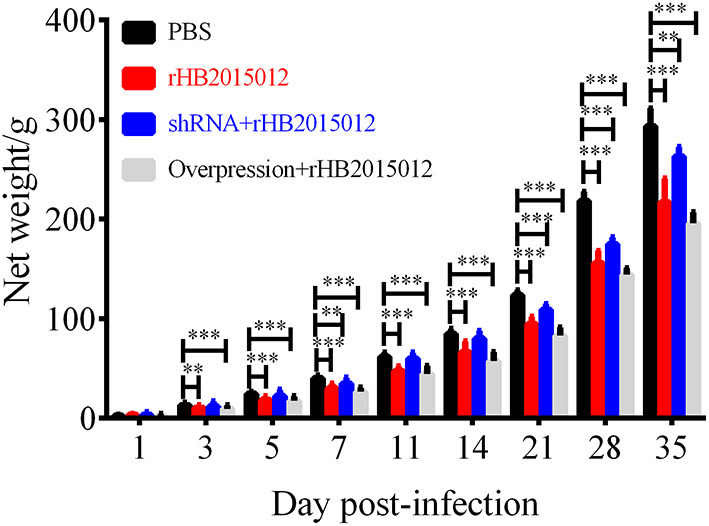
Net gain in animal body weight. There was a net increase in the weight of animals in the four groups. PBS group had the largest weight gain, different from others. After A20 overexpression, ALV-A rHB2015012 group had the smallest body weight. Growth was suppressed in A20-disrupted group, although body weight was higher than in rHB2015012 group. *P < 0.05, **P < 0.01, ***P < 0.001.

To explore the A20 effect on body weight after ALV-A infection, all chickens were observed and weighed at set time points, and the results are demonstrated in Figure 4. At 35 days of ALV-A infection, the average net weight gains of the negative, positive, shRNA, and overexpression groups were 293.4, 216.9, 263.0 and 195.7 g, respectively. These data suggest that A20 could regulate chicken growth after ALV-A infection.

### A20 overexpression promoted hepatomegaly

**Figure 5:**
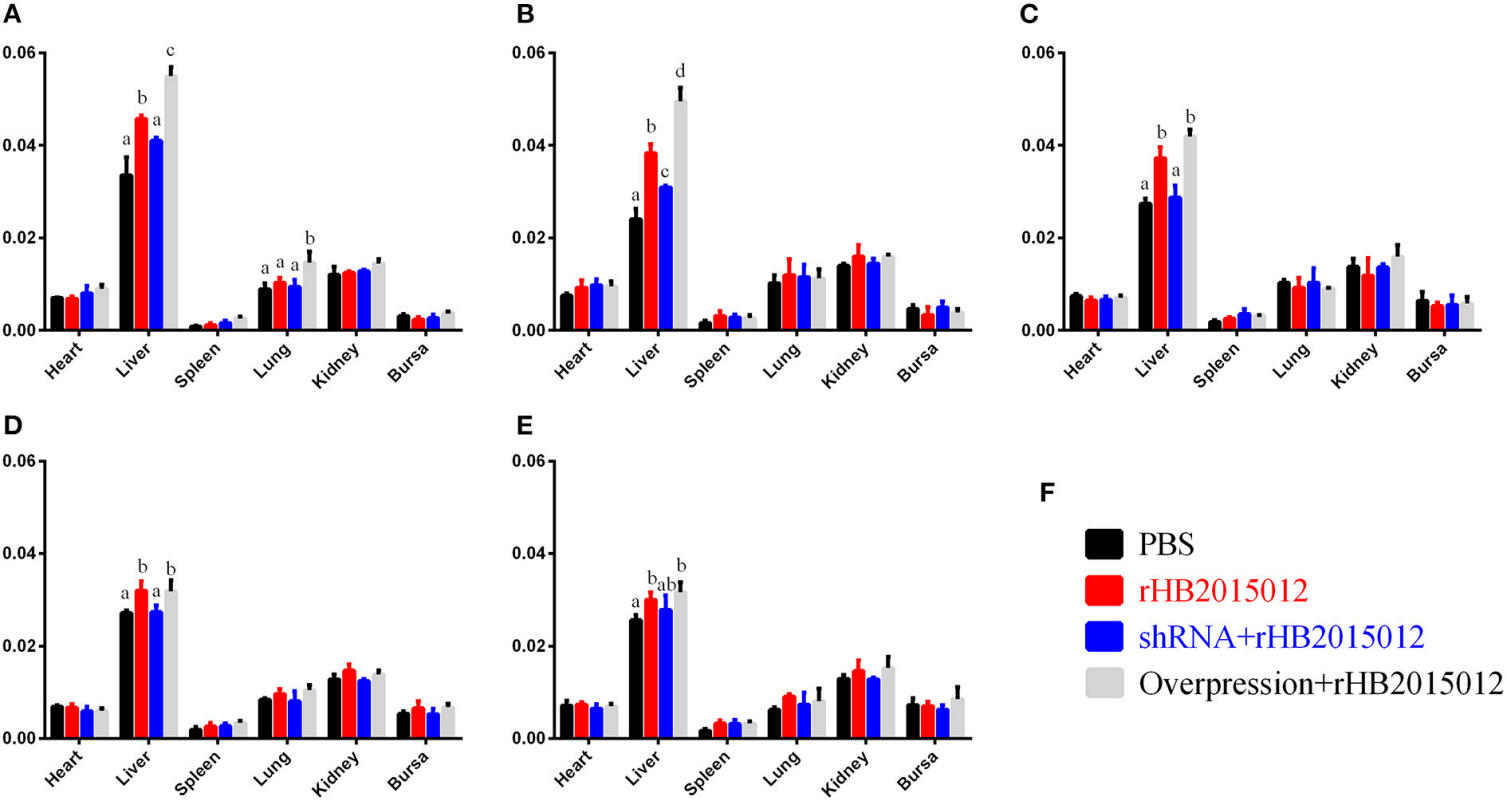
Animal experimental organ index. **(A–E)** 7-, 14-, 21-, 28- and 35-day organ index. ALV-A rHB2015012 infection in A20-overexpressing chickens promotes the occurrence of hepatomegaly symptoms but does not promote or inhibit heart, spleen, lung, kidney and bursa. **(F)** Animal experiment group legend. a, b, c, and d indicate differences, with the same letter indicating no difference and different letters indicating a difference.

To explore the A20 effect on organs and tissues after ALV-A infection, the organ index was calculated for the heart, liver, spleen, lung, kidney, and bursa. Surprisingly, differences were found in the liver but not in the spleen or kidney (Figure 5).

### A20 overexpression caused severe viremia

**Figure 6:**
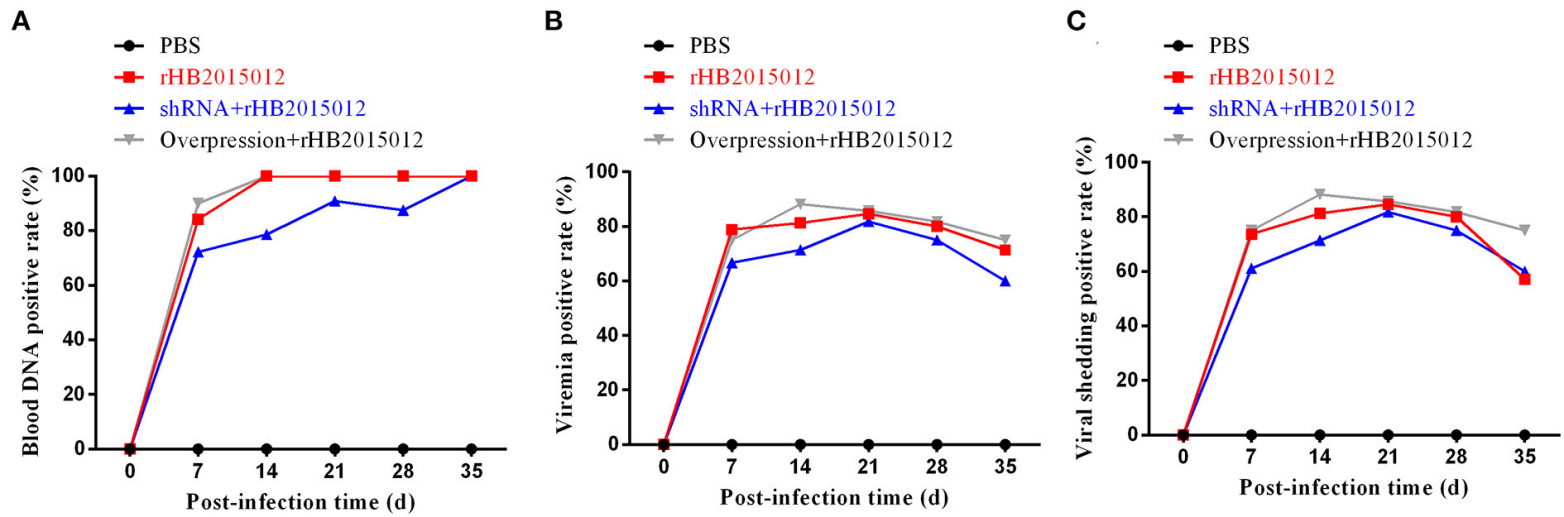
ALV-A infection status, viremia and viral shedding. **(A)** Identifying ALV-A infection by blood DNA PCR. **(B)** Viremia after ALV-A infection was detected by ELISA. **(C)** Detecting viral shedding after ALV-A infection by anal swab p27 group-specific antigen ELISA.

To monitor the occurrence of chicken viremia in the four groups, ALV-A/J/K multiplex PCR detection and p27 ELISA detection were performed on collected blood, and p27 ELISA detection was carried out on collected anal swabs, and the results are displayed in Figure 5. Blood DNA PCR (Figure 6A), viremia p27 ELISA (Figure 6B), and anal swab p27 ELISA (Figure 6C) revealed similar results; the overexpression group had the highest positive rate, followed by the positive group, and the shRNA group had the lowest.

### Antibody level in the A20 shRNA group was higher than A20 overexpression group

**Figure 7:**
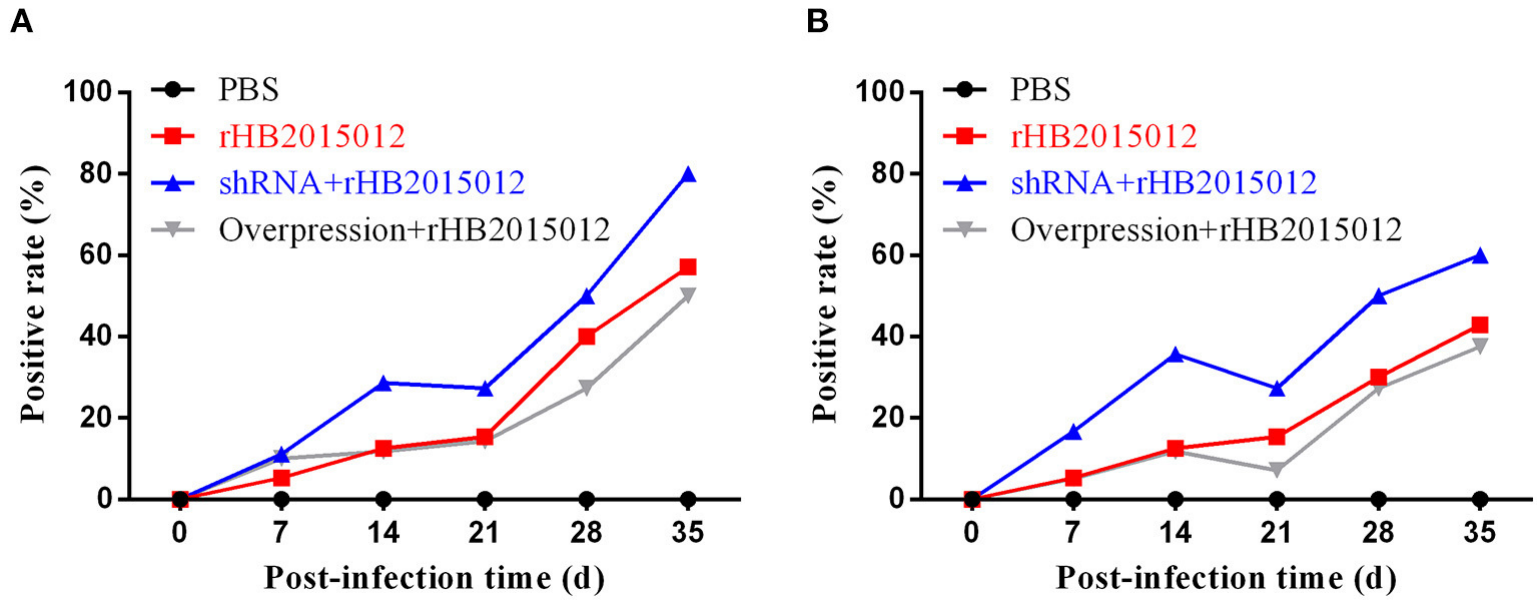
Antibody production after ALV-A infection. **(A)** Gp85 antibody production after ALV-A infection. **(B)** p27 antibody production after ALV-A infection.

To explore the effect of A20 expression on antibody production after ALV-A infection, collected blood was centrifuged to prepare serum for ELISA detection. On days 7, 14, 21, 28, and 35, the shRNA group had higher levels of Gp85 antibody than the positive group, and the levels in the overexpression group increased steadily (Figure 7A). Moreover, P27 antibody was detected. After 21 days, the antibody level in the shRNA group decreased slightly but was still higher than that in the positive and overexpression groups (Figure 7B).

### A20 overexpression promoted expression of envelope protein Gp85

**Figure 8:**
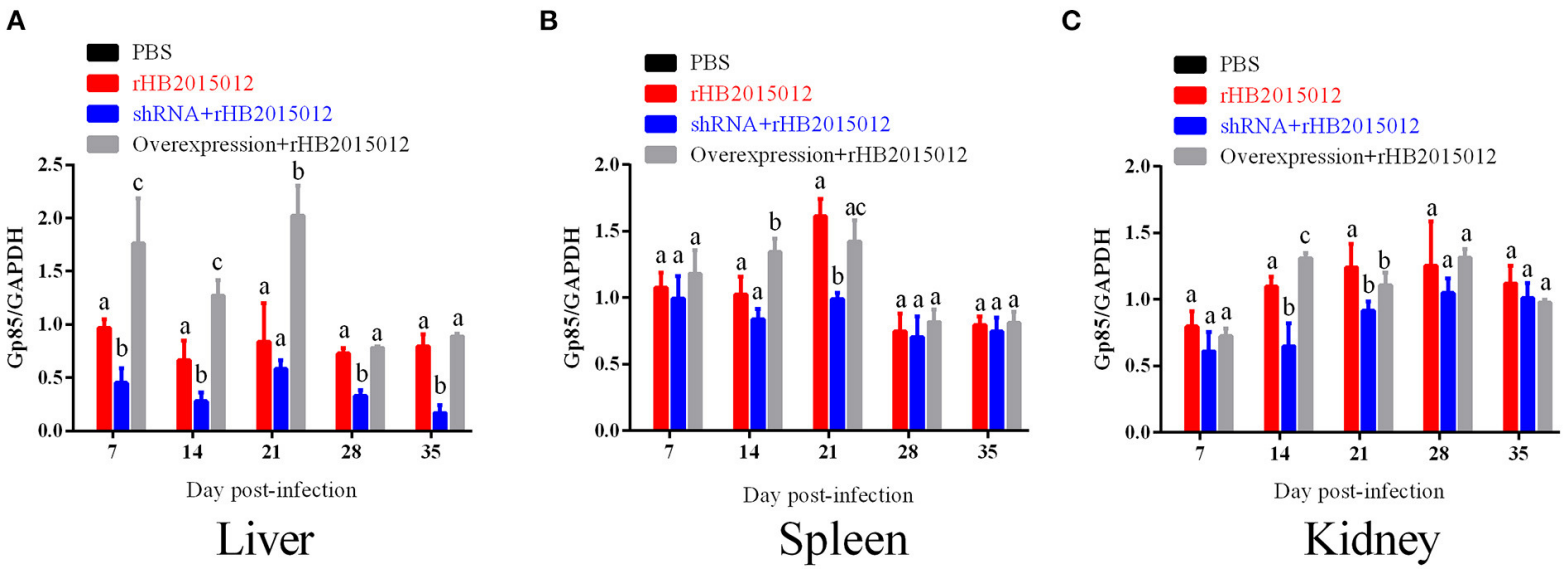
The viral loads in liver, spleen and kidney after ALV-A infection was determined by Western blotting. **(A)** Liver viral loads. **(B)** Spleen viral loads. **(C)** Kidney viral loads. a, b, and c indicate differences, with the same letter indicating no difference and different letters indicating a difference.

The expression of the envelope protein Gp85 in the liver, spleen, and kidney of ALV-A rHB015012 infected chickens was determined using Western blotting. Gp85 was differentially expressed in the liver between the A20 overexpression and interference groups (Figure 8A). Differential expression in the spleen at 14 and 21 days (Figure 8B). Differential expression in the kidney was observed only at 14 days (Figure 8C).

### A20 overexpression promoted viral replication *in vivo*

**Figure 9:**
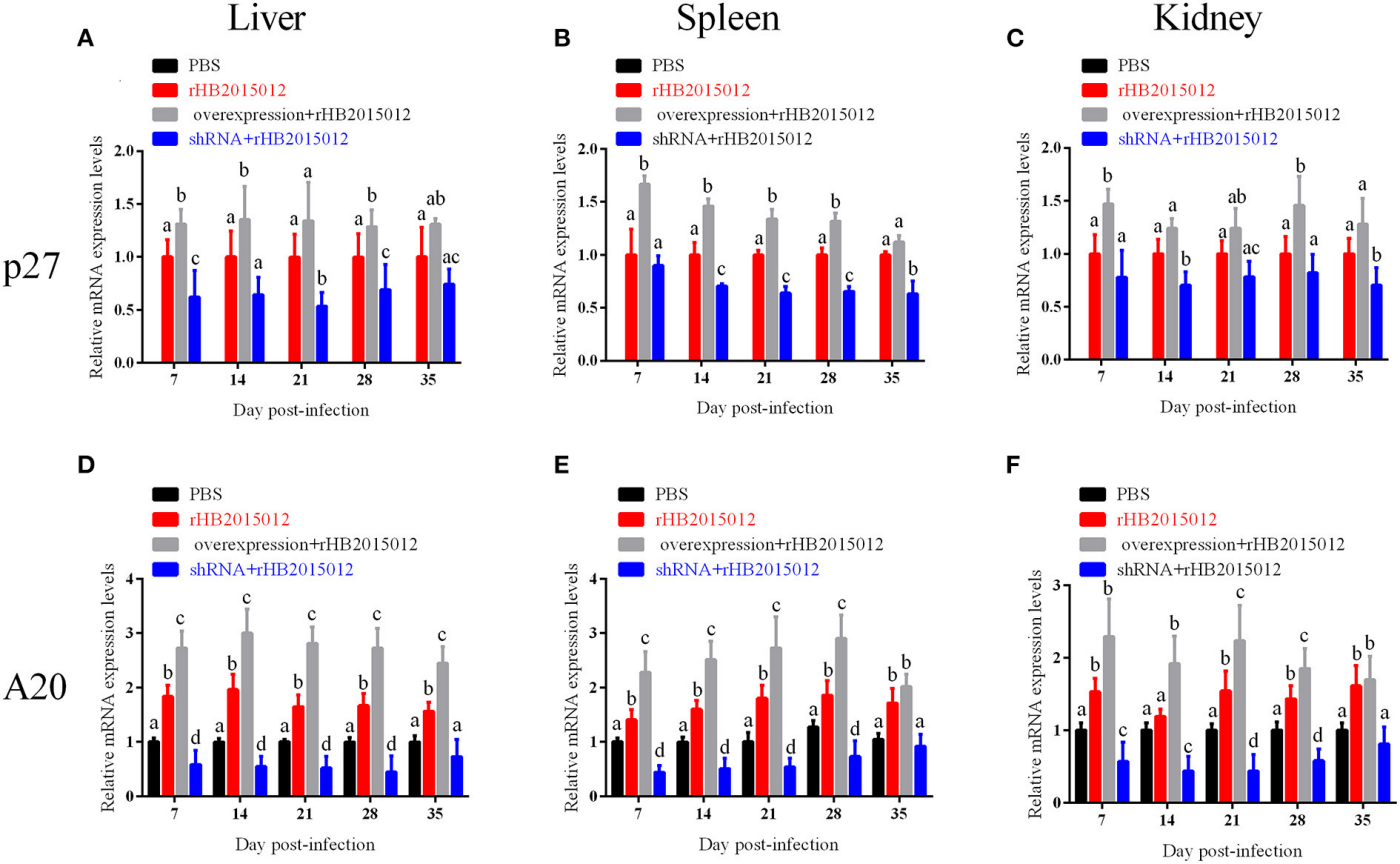
The viral loads in liver, spleen and kidney after ALV-A infection was determined by q-PCR. **(A–C)** Liver, spleen, and kidney viral loads. **(D–F)** Expression of A20 in liver, spleen, and kidney. a, b, c, and d indicate differences, with the same letter indicating no difference and different letters indicating a difference.

The time and extent of onset are closely related to the viral load. The viral loads of the four chicken groups were analyzed, and it was observed that the chickens in the negative group were uninfected. The results are displayed in Figure 9. The viral loads differed in the liver, spleen, and kidneys at 7, 14, 21, 28, and 35 days. Although the viral loads detected at different time points were incompletely consistent, they suggested that A20 overexpression promoted ALV-A replication *in vivo*.

### Pathological observation

**Figure 10:**
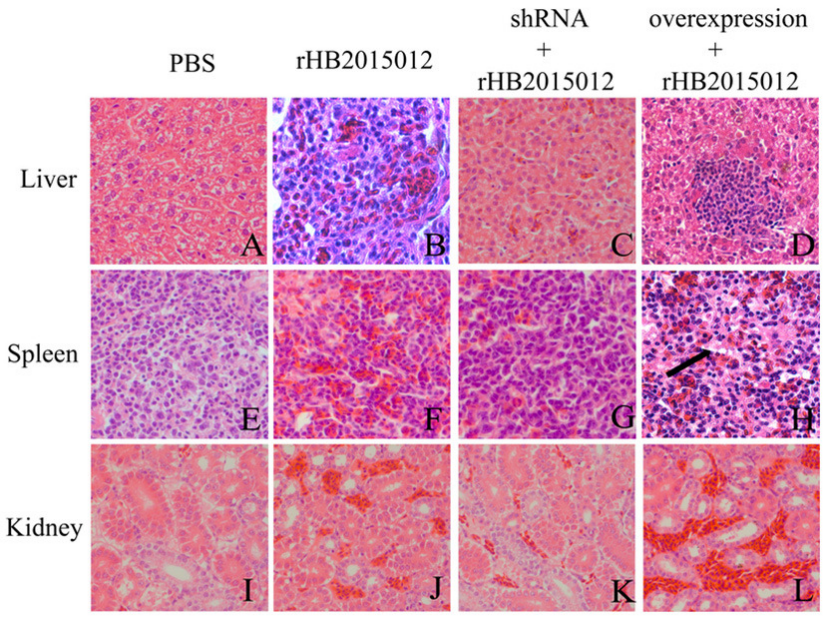
Pathological observations **(H,E)** of ALV-A infection at 100 days (400×). **(A,E,I)** Liver, spleen and kidney tissue sections in PBS group. ALV-A rHB2015012 infection caused liver congestion **(B)**, and tissue congestion was relieved after A20 expression was interfered with **(C)**, while A20 overexpression aggravated congestion with lymphocyte infiltration **(D)**. ALV-A rHB2015012 infection caused spleen congestion **(F)**, and tissue congestion was relieved after A20 expression was interfered with **(G)**, while A20 overexpression aggravated congestion with myeloma-like cells **(H)**. ALV-A rHB2015012 infection caused kidney congestion **(J)**, and tissue congestion was relieved after A20 expression was interfered with **(K)**, while A20 overexpression aggravated congestion **(L)**.

The collected tissues were prepared by slice preparation, and the results showed that the liver, spleen and kidney were congested 100 days after ALV-A rHB2015012 infection (Figures 10B,F,J), whereas the liver, spleen and kidney were congested after ALV-A rHB2015012 infection after A20 interference. All were in remission (Figures 10C,G,K), while ALV-A rHB2015012 infection following A20 overexpression resulted in massive lymphocytic infiltration in the liver (Figure 10D), myeloid tumor cells were found in the spleen (Figure 10H), and exacerbated renal Hyperemia (Figure 10L).

## Discussion

ALVs are classified into different subgroups based on their envelope proteins, and the tumorigenicity of each subgroup varies (28). ALV-A primarily causes classic lymphomas with an increasingly broad tumorigenic spectrum over time (28, 29). In 2015, we isolated an ALV-A strain (HB2015012) that caused lymphoma and myeloma. This finding was confirmed by animal regression experiments (30). The expanded tumorigenic spectrum of ALV-A can significantly affect the poultry industry.

A20 is involved in various diseases, including tumors. Adenovirus vectors have the advantages of a large vector capacity, a high transfection rate, and expressed proteins close to the natural conformation. Recombinant adenoviruses can infect several hosts, including dividing and non-dividing cells, but do not integrate with the host genome (26, 31, 32). Therefore, recombinant adenovirus was selected as the vector for A20 overexpression and shRNA interference in chickens.

The experimental animals were divided into four groups: negative, positive, overexpression, and shRNA. After inoculating chicken embryos with recombinant adenovirus, the negative and positive groups were injected with equal volumes of dilution (PBS) to eliminate the dilution effect on animals. When 1-day-old chicks were inoculated with ALV-A, the negative group received an equal dilution volume. After 3 days of ALV-A infection, the net growth of chickens began to differ, and the differences became more obvious with time (Figure 4). ALV infection usually causes enlargement of the liver, spleen, and kidney (28, 33, 34). Unexpectedly, only the liver's organ index was higher than that of the negative group, but there was no difference between the spleen and kidney (Figures 5A–E). This may be related to the nature of the virus or the chicken breed.

ALVs are retroviruses that integrate the viral genome into the host genome after vertical transmission (35). The blood DNA PCR results demonstrated that the positive rate of the positive and overexpression groups was 100% at 14 days, while that of the shRNA group reached 100% at 35 days, indicating that A20 may play a role in ALV-A replication (Figure 6A). Blood and anal swab p27 ELISA results were similar to blood DNA PCR results, although the positive rate did not reach 100% (Figures 6B,C). Although there may be endogenous virus interference in serum samples using p27 ELISA, the fact that it can reflect the presence of when combined with other methods. ALV infection can cause immunosuppression, affecting antibody production and vaccine efficacy (33, 35–38). Gp85 antibody can neutralize the virus, whereas the p27 antibody reflects viral replication. The results showed that the positive rate of antibody production increased after A20 expression was reduced (Figures 7A,B). The viral load determines the onset time and degree of onset (33). In this study, Western blotting (Figure 8) was used to measure the expression of envelope protein Gp85, and qPCR (Figure 9) was used to measure the viral load. Since many factors affect Western blotting, this method is as inaccurate as qPCR. However, it can reflect the trend of facts; A20 overexpression could promote ALV-A replication *in vivo*. Because adenovirus existence has a certain time limit, A20 overexpression and interference did not work after 28 days by Western blotting (Supplementary Figures 2D,E). However, the data before 28 days may still indicate that A20 can promote ALV-A replication *in vivo* (Supplementary Figures 2A–C). Q-PCR showed that the adenovirus lost its effect at 35 days, but the viral load was still different between the overexpression and interference groups (Figures 9A,E). Pathological profiling revealed that A20-overexpressing chickens exhibited severe liver, spleen, and kidney lesions (Figure 10).

In conclusion, we constructed A20 recombinant adenovirus inoculated animals and evaluated the A20 effect on ALV-A in terms of body weight, viremia, antibody, and viral load. These results confirmed that A20 plays a role in promoting ALV-A replication. This study lays the foundation for future research on the tumorigenic mechanism of ALV-A mediated by A20.

## Data availability statement

The raw data supporting the conclusions of this article will be made available by the authors, without undue reservation.

## Ethics statement

The animal study was reviewed and approved by Yangtze University.

## Author contributions

XC and CF designed the study. XC conducted the experiments and analyzed the data. YuyY, XL and JL provided funds. XC, XW, YuxY, CF, JL, XL, and YuyY participated in writing this article. All authors contributed to the article and approved the submitted version.

## Funding

This work was supported by the National Nature Science Foundation of China (No. 31972646), Scientific Research Project of the Education Department of Hubei Province (B2019028), Scientific Research Project of Jingzhou City (2020CB21-31), and Hubei Nature Province Science Foundation of China (2021CFB173).

## Conflict of interest

The authors declare that the research was conducted in the absence of any commercial or financial relationships that could be construed as a potential conflict of interest.

## Publisher's note

All claims expressed in this article are solely those of the authors and do not necessarily represent those of their affiliated organizations, or those of the publisher, the editors and the reviewers. Any product that may be evaluated in this article, or claim that may be made by its manufacturer, is not guaranteed or endorsed by the publisher.

## Supplementary material

Supplementary Figure 1 Validation of 40-week-old chicken liver RNA-seq sequencing and qRT-PCR results.

Supplementary Figure 2 Western blotting tissue viral load assay. Tissue viral loads in **(A)** 7-day-old, **(B)** 14-day-old, **(C)** 21-day-old, **(D)** 28-day-old, and **(E)** 35-day-old chickens.
