# Supplementary figures and images for: Enhanced pathogenicity by up-regulation of A20 after avian leukemia subgroup a virus infection
Source: Front Vet Sci. 2022 Nov 14;9:1031480. doi: 10.3389/fvets.2022.1031480 (PMC9702354; doi:10.3389/fvets.2022.1031480)

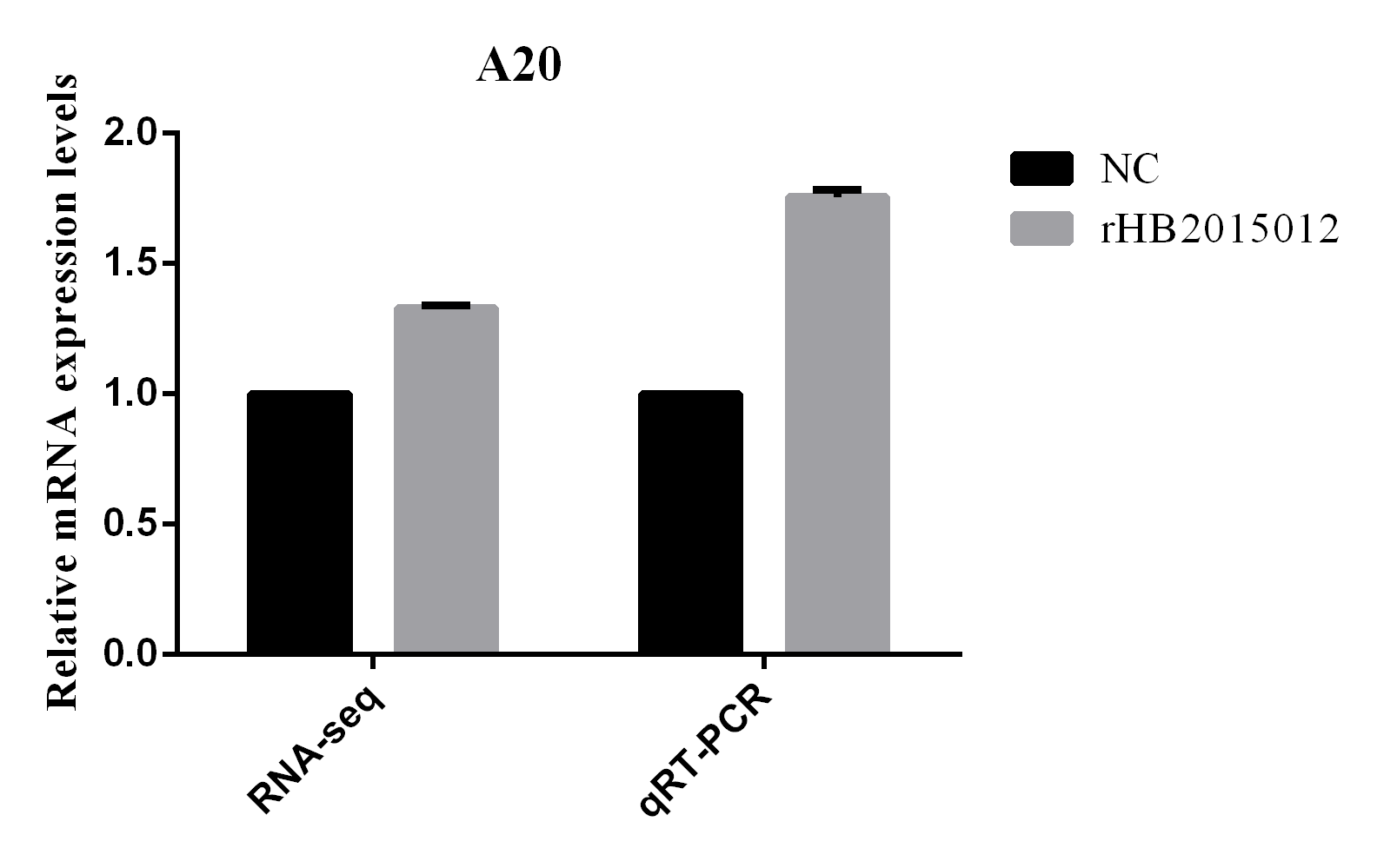

Supplement: Supplementary Figure 1 — Validation of 40-week-old chicken liver RNA-seq sequencing and qRT-PCR results. [file Image_1.TIF]

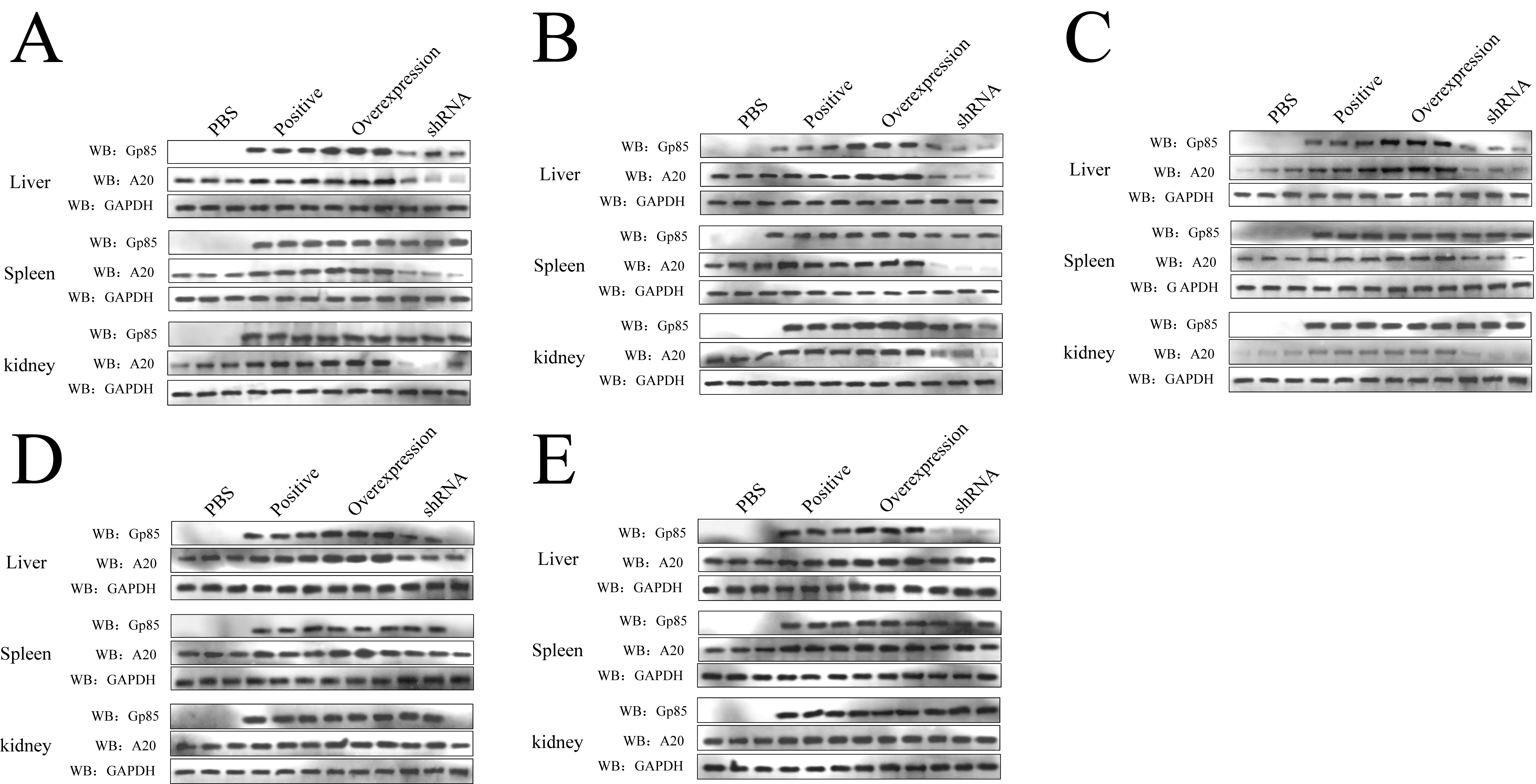

Supplement: Supplementary Figure 2 — Western blotting tissue viral load assay. Tissue viral loads in (A) 7-day-old, (B) 14-day-old, (C) 21-day-old, (D) 28-day-old, and (E) 35-day-old chickens. [file Image_2.TIF]
